# The P2X_7_ receptor regulates proteoglycan expression in the corneal stroma

**Published:** 2012-01-18

**Authors:** Courtney Mankus, Cheryl Chi, Celeste Rich, Ruiyi Ren, Vickery Trinkaus-Randall

**Affiliations:** 1 ECI Biotech, Worcester, MA; 2 Department of Ophthalmology, Boston University School of Medicine, Boston, MA; 3 Department of Biochemistry, Boston University School of Medicine, Boston, MA

## Abstract

**Purpose:**

Previously, the authors demonstrated that the lack of the P2X_7_ receptor impairs epithelial wound healing and stromal collagen organization in the cornea. The goal here is to characterize specific effects of the P2X_7_ receptor on components of the corneal stroma extracellular matrix.

**Methods:**

Unwounded corneas from P2X_7_ knockout mice (*P2X_7_*^−/−^) and C57BL/6J wild type mice (WT) were fixed and prepared for quantitative and qualitative analysis of protein expression and localization using Real Time PCR and immunohistochemistry. Corneas were stained also with Cuprolinic blue for electron microscopy to quantify proteoglycan sulfation in the stroma.

**Results:**

*P2X_7_*^−/−^ mice showed decreased mRNA expression in the major components of the corneal stroma: collagen types I and V and small leucine-rich proteoglycans decorin, keratocan, and lumican. In contrast *P2X_7_*^−/−^ mice showed increased mRNA expression in lysyl oxidase and biglycan. Additionally, we observed increases in syndecan 1, perlecan, and type III collagen. There was a loss of perlecan along the basement membrane and enhanced expression throughout the stroma, in contrast with the decreased localization of other proteoglycans throughout the stroma. In the absence of lyase digestion there was a significantly smaller number of proteoglycan units per 100 nm of collagen fibrils in the *P2X_7_*^−/−^ compared to WT mice. While digestion was more pronounced in the WT group, double digestion with Keratanase I and Chondroitinase ABC removed 88% of the GAG filaments in the WT, compared to 72% of those in the *P2X_7_*^−/−^ mice, indicating that there are more heparan sulfate proteoglycans in the latter.

**Conclusions:**

Our results indicate that loss of P2X_7_ alters both the expression of proteins and the sulfation of proteoglycans in the corneal stroma.

## Introduction

P2X_7_ is one of seven known ion-gated purinergic receptors. It is naturally activated by ATP, which is released in injury or stress conditions, leading to an increase in intracellular calcium. The receptor can also be activated by BzATP, a potent synthetic agonist that is specific for the receptor [1-3]. The P2X_7_ receptor plays various signaling roles, depending on the cell type. For example, P2X_7_ induces apoptosis in many cells through lethal increases of intracellular calcium [1,3]. In other cell types, such as macrophages or microglia, P2X_7_ activates the mitogen-activated protein kinase (MAP) kinase/extracellular signal-regulated kinase (ERK)1/2 signaling pathway to promote cell growth and proliferation [1,4,5]. In the cornea, P2X_7_ has been shown to facilitate wound healing and epithelial cell migration, and regulate collagen organization in the stroma [6].

Type I collagen, a major extracellular matrix protein of the cornea, is synthesized as precursor molecules that undergo post-translational modifications and are arranged into fibrillar arrays that become cross-linked by lysyl oxidase [7-9]. Cross-linking increases the tensile strength of collagen and is necessary to withstand constant stresses [7]. In the stroma, which constitutes approximately 90% of the cornea’s thickness, collagen is usually organized into lamellae. The lamellae are arranged at an angle, often nearly orthogonal to each other [9,10]. An individual lamella consists of many collagen fibrils arranged in parallel. In addition, fibrils are detected inserting into Bowman’s membrane in the anterior stroma [11]. This precise organization is extremely important to the transparent properties of the cornea [7,10,12].

Previously, we showed that the lack of P2X_7_ in the cornea affected stromal organization and expression of collagen and associated proteins [6]. Lamellar organization changed from a nearly orthogonal arrangement to a swirling pattern. Individual fibrils were thinner and interfibrillar spacing was greater, leading to increased width of lamellae and separation into layers in the mid and posterior stroma.

Collagen alignment is directly related to expression of proteoglycans, which bind to the structures and help organize the collagen into fibrillar arrays [9,13]. Though type I collagen is the predominant component of the stroma, type V collagen and specific proteoglycans are important in regulating fibril diameter [7,14,15]. Each proteoglycan comprises a core protein attached to varying numbers of glycosaminoglycan side chains, whose sulfated moieties hydrate the cornea and contribute to regulation of interfibrillar spacing [9,13,16-18]. For these reasons, small leucine-rich proteoglycans (SLRPs) are important to the transparency and refractile properties of the cornea. Decorin and biglycan have one and two glycosaminoglycan (GAG) side chains, respectively. Decorin also binds type I collagen to the carboxyl termini of type XII and type XIV collagen, members of the fibril-associated collagens with interrupted triple helices (FACIT) family found in the cornea [9,19-21]. Keratocan and lumican contain keratan sulfate side chains, and are important for maintaining the spacing of collagen fibrils and the curvature of the cornea [13,16,18,22]. Keratocan expression is specific to the cornea [13,22]. Additionally, decorin prevents lateral expansion of collagen fibrils and lumican promotes lengthwise growth [13,14,17,23].

The heparan sulfate proteoglycans (HSPGs), syndecan and perlecan, are important for maintenance of corneal integrity. Perlecan’s various functions include mediating cell adhesion by binding to type IV collagen and laminin, components of the basement membrane, and binding to growth factors that mediate cell migration and proliferation [24]. Syndecan acts through α9 integrin localization in corneal keratinocytes to mediate cell migration and proliferation in wound closure [25].

The purpose of this study was to explain the causes of stromal variation due to P2X_7_ deficiency. We investigate the altered collagen properties in *P2X_7_*^−/−^ mice and examine the role of the P2X_7_ receptor in maintaining corneal structure and function. Our data demonstrate that the expression and localization of proteoglycans and related proteins are altered in P2X_7_-deficient corneas, indicating a regulatory role for P2X_7_ in the corneal stroma. We hypothesize that P2X_7_ plays a critical role in the development and/or regenerative capacity of the corneal stroma.

## Methods

### Materials

P2X_7_ null mice (*P2X_7_*^−/−^) strain B6.129P2-P2rx7^tm1GaB^/J and wild-type mice (WT) strain C57BL/6J were acquired from Jackson Laboratories (Bar Harbor, ME) 5 weeks postnatal and were acclimated for 16 days. The polyclonal antibody against keratocan was from Santa Cruz Biotechnology, Inc. (Santa Cruz, CA), the polyclonal decorin and lumican antibodies were purchased from R&D Systems, Inc. (Minneapolis, MN) and the monoclonal antibody against perlecan was from Millipore (Billerica, MA). SlowFade antifade reagent, To-Pro 3AM, Alexa Fluor-conjugated secondary antibodies, TRIzol reagent, DNasel, Maloney Murine Leukemia Virus reverse transcriptase (MMLV-RT), random hexamers, and RNaseH were purchased from Invitrogen (Carlsbad, CA). RNase inhibitor was purchased from Roche Applied Science (Indianapolis, IN). TaqMan Gene Expression Assays were purchased from Applied Biosystems (Foster City, CA). Keratanase I, Heparinase I and III were from Seikagaku (Tokyo, Japan), and Chondroitinase ABC was from Sigma (St. Louis, MO). Cuprolinic blue and other supplies for electron microscopy were purchased from Electron Microscopy Sciences (Hatfield, PA). Routine chemicals were purchased from Qiagen (Valencia, CA), American Bioanalytical (Natick, MA) and Fisher Scientific (Waltham, MA).

### Animals

Animals were used in accordance with international standards for animal treatment established by the National Institutes of Health and ARVO. *P2X_7_*^−/−^ and WT mice were euthanized using carbon dioxide asphyxiation and cervical dislocation. Death was confirmed by observing the lack of a toe-pinch response. For RNA extraction, the corneal epithelium was removed from the eye using a sterile surgical blade. The de-epithelialized corneas from four mice were pooled and frozen at −80 °C.

### Real time polymerase chain reaction (RT–PCR)

Each target was amplified from three independent pools of six stromas each, obtained from mice of the same litter as described [6]. Experiments were performed on three independent samples of each group. Briefly, RNA was extracted from homogenized corneal stromas using TRIzol as per the manufacturer’s guidelines, and total RNA concentration was determined with a spectrophotometer. RNA was incubated with DNase I and 1 U/μl RNase inhibitor to remove any contaminating genomic DNA. Then, reverse transcription was performed with 100 U/μl MMLV-RT, 10 ng/μl random primers, 0.5 nM dNTP mixture, and 10 mM DTT in 10× first strand reaction buffer. Negative control reactions were assembled without MMLV-RT. The cDNA produced was treated with RNaseH. Real Time PCR was performed using an ABI 7300 cycler (Applied Biosystems, Foster City, CA). The TaqMan Eukaryotic 18S rRNA Endogenous control assay was used in addition to the TaqMan gene expression assays indicated in Table 1. The cycling parameters were as follows: an initial 10 min incubation at 95 °C, followed by 45 cycles of 95 °C for 15 s and 60 °C for 1 min. The ΔΔCt method was used to determine the relative expression of each transcript, normalized to the 18S ribosomal subunit. Comparisons to determine statistical significance between WT and *P2X_7_*^-/-^ were made using Student’s *t*-test.

**Table 1:** TaqMan^®^ Gene Expression Assays. Real Time PCR was performed using 1 µl of each TaqMan^®^ Gene Expression Assays from Applied Biosystems (Foster City, CA) to detect and quantify the corresponding mRNA target.

**Target**	**Applied Biosystems TaqMan® Gene Expression Assay**
β-actin	Mm026109580_g1
Collagen α1(I)	Rn00801649_g1
Collagen α1(III)	Mm00802331_m1
Collagen α1(V)	Mm00489842_m1
Collagen α1(XII)	Mm00483425_m1
Collagen α1(XIV)	Mm00805269_m1
Lysyl Oxidase	Rn00566984_m1
Lysyl Oxidase Like-2	Mm00804740_m1
Lysyl Oxidase Like-3	Mm00442953_m1
Lysyl Oxidase Like-4	Mm00446385_m1
Decorin	Mm00514535_m1
Keratocan	Mm00515230_m1
Biglycan	Mm00455918_m1
Lumican	Mm01248292_m1
Perlecan	Mm01181165_m1
Syndecan 1	Rn00564662_m1
Syndecan 1	Rn00564662_m1
Syndecan 4	Rn00561900_m1

### Immunohistochemistry and image acquisition

Eyes were enucleated, placed cornea side down in Optimal Cutting Temperature (OCT) media and flash frozen. Six micron sections were cut and the slides were stored at −20 °C. Experiments were performed on three independent samples of each group.

The slides containing cryostat sections were dried at room temperature for 1.5 h. Sections stained with anti-keratocan were pre-digested with Keratanase I. Sections stained with anti-perlecan were pre-digested with Heparinase I and Heparinase III. Following digestion, sections were fixed in 4% paraformaldehyde for 30 min. Slides were rinsed with PBS and blocked with 3% BSA in PBS. The slides were dried around the sections and incubated with primary antibody diluted in 1% BSA in PBS for 1 h. Negative controls were incubated with either 1% BSA in PBS alone or non-immune IgG from the host animal. The slides were then rinsed 3 times with PBS, blocked again with 3% BSA in PBS for 30 min, and incubated with Alexa Fluor-conjugated secondary antibody diluted in 1% BSA in PBS for 1 h. After washing, slides were incubated for 15 min with To-Pro 3AM diluted 1:1,000 in PBS. Slides were washed, covered with Slowfade reagent, and coverslipped [6].

Sections were imaged using a Zeiss 200M LSM 510 confocal microscope (Zeiss, Thornwood, NY) [6,26]. The negative control samples incubated with secondary antibody alone were imaged first to eliminate fluorescent signal from non-specific binding of the secondary antibody. For controls, the detector gain and amplifier offset were set at the highest levels possible without obtaining fluorescence, and the levels of the detector gain and amplifier offset were maintained when imaging experimental sections, as any signal obtained above these levels represent specific fluorescence. Image analysis was performed using the Zeiss Image Processing software.

### Cuprolinic blue and electron microscopy

Cuprolinic blue was used to detect sulfated proteoglycans. Four corneas from two 9-week-old WT and *P2X_7_*^−/−^ mice were stained with 0.2% Cuprolinic blue (in buffer containing 0.3 M MgCl_2_) for 24 h [27]. Selective polysaccharidases were used to identify and quantitate GAGs and proteoglycan core proteins. Enzymes were tested for activity and specificity using highly purified GAG standards (Seikagaku, Tokyo, Japan). Concentrations of standards were determined before and after digestion with specific enzymes using the dimethylene blue (DMB) assay as described [28,29]. Briefly, purified proteoglycans were subjected to digestion for 3 h at 37 °C in 40 mM Tris-HCl. The pH of the digestion was adjusted to the optimum for either Chondroitinase ABC (1.0 unit/ml, pH 8.0), or Keratanase I (0.01 unit/ml, pH 5.9) before staining with Cuprolinic blue. Alternatively, the tissue was digested with Keratanase  I followed by Chondroitinase ABC before staining with Cuprolinic blue. The corneas were then post-fixed in Karnovsky’s fixative and stained with Cuprolinic blue and serially dehydrated before processing for TEM. Corneas were embedded in Epon-Araldite, sectioned serially onto carbon-coated copper grids, and stained with 4% water based uranyl acetate. Images were taken on a Philips 300 TEM (Eindhoven, The Netherlands). The number and periodicity of Cuprolinic-positive filaments were determined using NIH ImageJ software. Results were averaged and expressed as ± standard error of the mean (SEM) and one-way ANOVA followed by Tukey’s post-hoc test was performed.

## Results

### Expression of collagen and associated protein transcripts in *P2X_7_*^−/−^ stromas

**Figure 1:**
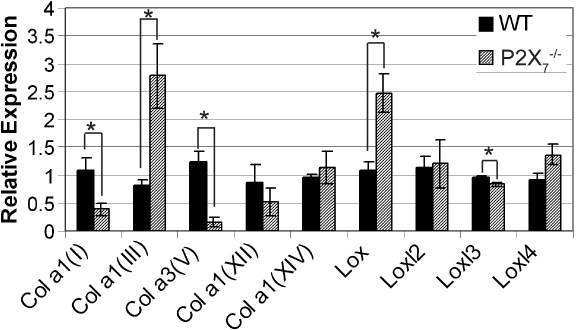
*P2X_7_*^−/−^ stromas show altered expression of collagen and related proteins. Real Time PCR results±SEM of at least three independent experiments were calculated using the ΔΔCt method and normalized to 18S rRNA. All sample results are presented relative to the median WT sample, normalized to 1. Negative controls were performed without reverse transcriptase. * p<0.03, Student’s *t*-test.

We studied the composition of corneal stromas in WT and *P2X_7_*^−/−^ mice using RT–PCR to detect transcripts of collagen, lysyl oxidase (*LOX*), and lysyl oxidase-like proteins (*LOXl*). There was a significant decrease in the two most highly expressed stromal collagen types, collagen α1(I) and collagen α3(V), in *P2X_7_*^−/−^ mice when compared with WT (p≤0.05). Expression of collagen α1(III), which is a marker of the wounded cornea and corneal scarring and is not usually detected in the unwounded stroma, was significantly increased in *P2X_7_*^−/−^ mice compared to WT (p≤0.05) (Figure 1). There was no significant difference in expression of collagen α1(XII) or collagen α1(XIV), members of the FACIT collagen family (Figure 1).

Expression of *LOX* and related *LOXl* was examined to determine if alteration in transcript levels contribute to smaller collagen fibril diameters in *P2X_7_*^−/−^ mouse stromas [6]. Lysyl oxidase expression in *P2X_7_*^−/−^ stromas was significantly greater than in WT stromas (Figure 1). While *LOXl3* expression in *P2X_7_*^−/−^ stromas was less than in WT stromas, there was no difference in *LOXl2* and *LOXl4* expression. Neither sample expressed detectable amounts of *LOXl1*.

### Expression of stromal proteoglycan transcripts in *P2X_7_*^−/−^ mice

The expression of proteoglycans was studied in *P2X_7_*^−/−^ corneas to determine potential causes of altered collagen lamellae organization in the stroma. Using RT–PCR, we determined the relative expression of proteoglycan core proteins: keratocan, decorin, lumican, biglycan, syndecans 1 and 4, and perlecan. Relative expression of β-actin (*Actb*) mRNA was unchanged between WT and *P2X_7_*^−/−^ mice.

**Figure 2:**
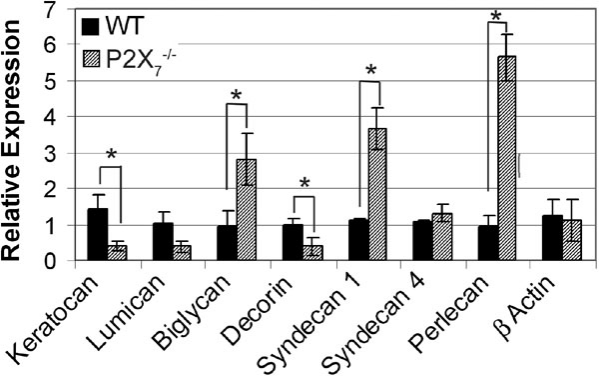
Altered expression of proteoglycan core proteins in *P2X_7_*^−/−^ stromas. Real Time PCR results±SEM of at least three independent experiments were calculated using the ΔΔCt method and normalized to 18S rRNA. All sample results are presented relative to the median WT sample, normalized to 1. Negative controls were performed without reverse transcriptase. * p<0.05, Student’s *t*-test.

There were reductions in the expression of 3 out of 4 small leucine-rich proteoglycans (SLRPs) in *P2X_7_*^−/−^ stromas (Figure 2). Relative expression of keratocan decreased from 1.4 in WT to 0.41 in *P2X_7_*^−/−^ and decorin decreased from 0.99 to 0.38 (p≤0.05). The expression of lumican decreased in *P2X_7_*^−/−^, from 1.02 in WT to 0.39 in the knockout but the difference was not significant according to Student’s *t*-test (p=0.07). Compared to decorin, the expression of biglycan was higher in the *P2X_7_*^−/−^ stromas, where relative expression was 2.8 compared to 0.96 in WT stromas (p≤0.05; Figure 2). This may be the result of a compensatory mechanism due to the decreased expression in decorin, as was previously reported in decorin null mice [9,12,13].

Perlecan has been found in low levels in the normal stroma and is increased upon injury [18,30,31]. In the unwounded *P2X_7_*^−/−^ stroma, both syndecan 1 and perlecan demonstrated increased expression compared to the WT (Figure 2). As expected, there was no difference in expression of syndecan 4. These data, along with the results showing decreased expression of decorin and keratocan, suggest that the lack of P2X_7_ holds the cornea in a pseudo-wounded state, as syndecan 1 and perlecan are markers of corneal wounding and scarring.

### Changes in localization of keratan sulfate proteoglycans and decorin

**Figure 3:**
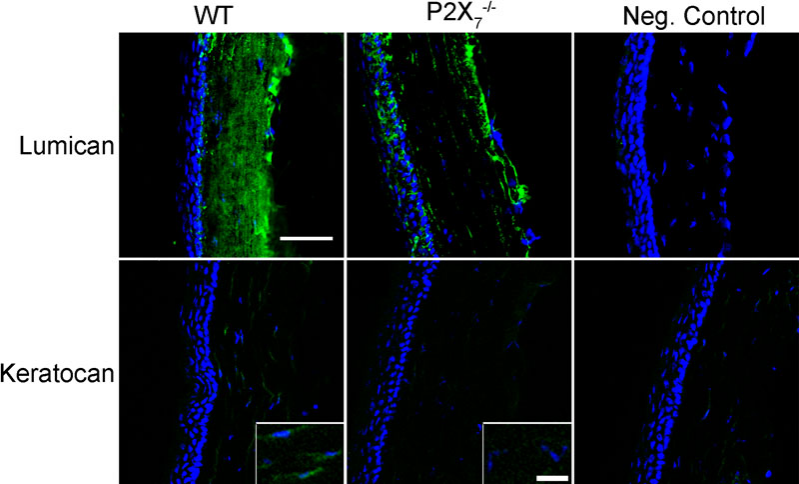
KSPG localization is altered in *P2X_7_*^−/−^ stromas. Frozen corneas were sectioned and stained with antibodies specific for keratocan and lumican, followed by FITC-conjugated IgG (green) and counterstained with To-Pro 3AM for nuclei (blue). Negative controls were incubated with secondary antibody only and counterstained with To-Pro 3AM. Images are representative of three independent experiments. Keratocan (inset): enlarged regions from central stroma with enhanced signal to show detail of localization. Scale bar: 10 μm.

We examined proteoglycan localization using immunohistochemistry. Lumican was detected in a similar expression pattern in both *P2X_7_*^−/−^ and WT stromas, apparently aligned along the longitudinal arrays of collagen. However, there were large areas that lacked lumican expression in the *P2X_7_*^−/−^ stromas, which were not observed in the WT stromas (Figure 3). Additionally, in the *P2X_7_*^−/−^ stromas the lumican was more intense in the posterior region than the anterior and middle regions. While there was only minimal detection of keratocan in the WT (Figure 3, inset), it was not detectable in the *P2X_7_*^−/−^ stromas, consistent with decreased keratocan mRNA expression in *P2X_7_*^−/−^ stromas (Figure 2).

**Figure 4:**
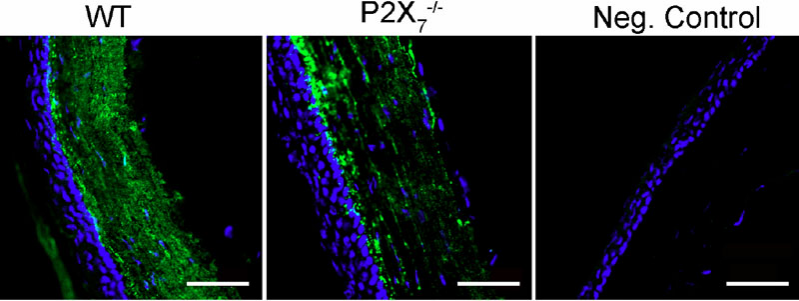
Decorin localization is altered in *P2X_7_*^−/−^ stromas. Frozen corneas were sectioned and stained with antibody against decorin, FITC-conjugated IgG, and To-Pro 3AM. Negative controls were incubated with non-immune IgG instead of primary antibody, and To-Pro 3AM. Images are representative of three independent experiments. E=Epithelium, S=Stroma. Scale bar: 50 μm.

The localization of decorin was similar in WT and *P2X_7_*^−/−^ stromas. However, the staining was reduced in the *P2X_7_*^−/−^ and large spaces lacking the presence of detectable decorin were observed only in the knockout tissue (Figure 4). This overall decrease in decorin protein correlated with the decrease in decorin mRNA (Figure 2).

### Changes in glycosaminoglycan sulfation along collagen fibrils

**Figure 5:**
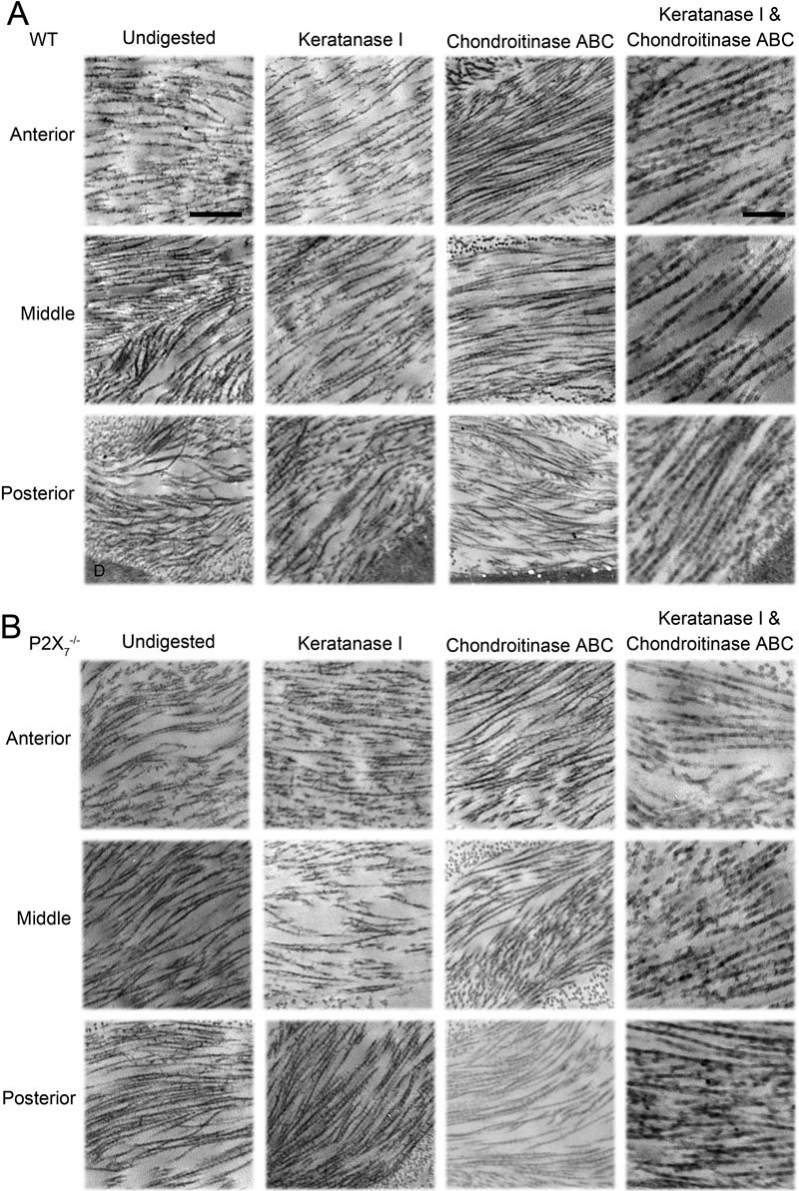
Electron micrographs of electron dense filaments stained with Cuprolinic blue on stromal collagen. Samples of **A**: WT and **B**: *P2X_7_*^−/−^ corneas were digested with either Keratanase I, Chondroitinase ABC, or both, before Cuprolinic blue staining and imaging with TEM. Undigested and stained tissue are included. The scale bar seen in the undigested column represents 500 nm, and applies to all images in the Undigested, Keratanase I, and Chondroitinase ABC columns. The scale bar seen in the Keratanase I and Chondroitinase ABC columns represent 200 nm and applies to images in that column only. Descemet’s membrane is marked with “D” and is visible in the WT posterior images.

**Figure 6:**
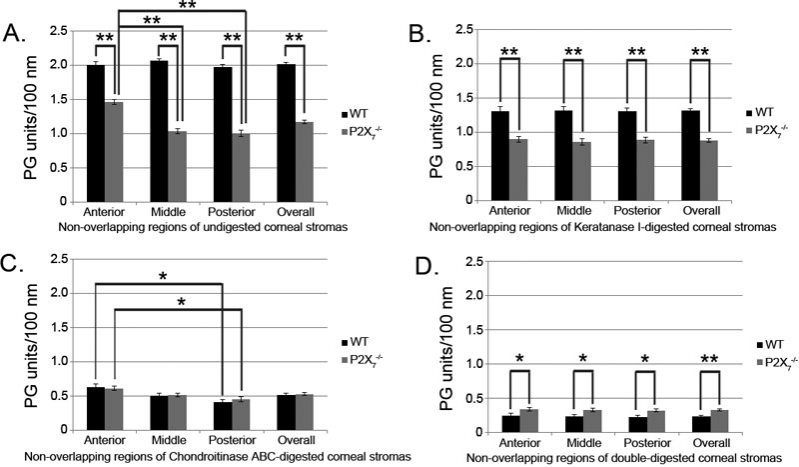
Sulfation of glycosaminoglycans in WT and *P2X_7_*^−/−^ corneal stromas. Collagen fibril length was measured, and the number of proteoglycan (PG) units along that length was counted for each non-overlapping region (Anterior, Middle, Posterior) and summed for the entire cornea (Overall). Results were normalized to number of PG units per 100 nm collagen for **A**: Undigested corneas, **B**: Corneas digested with Keratanase I, **C**: Corneas digested with Chondroitinase ABC, and **D**: Corneas digested with both Keratanase I and Chondroitinase ABC. A minimum of 75 measurements were performed for each region of each group and digestion condition, and results were averaged and presented as ±SEM ** p<0.0001 and * p<0.05, one-way ANOVA followed by Tukey’s post-hoc test.

Cuprolinic blue staining in the presence or absence of lyase digestion and electron microscopy were performed to quantify the sulfated GAG moieties associated with collagen filaments. The sulfated GAGs are depicted as electron dense filaments [6,32,33]. Images of three regionally distinct populations of collagen fibrils were taken in the presence or absence of lyases (Figure 5). In the undigested sections, there were a smaller number of proteoglycan units per 100 nm of collagen fibril in the *P2X_7_*^−/−^ when compared to WT (Figure 6A), indicating a decrease in proteoglycan sulfation. While the distribution of sulfated GAGs throughout the WT stroma was homogeneous, there was a higher density of electron dense filaments or sulfated GAGs observed in the anterior portion of *P2X_7_*^−/−^ corneas than the middle or posterior portions.

To determine the relative presence of glycosaminoglycan subtypes, samples were digested with Keratanase I and/or Chondroitinase ABC. Keratanase I reduced the number of filaments per 100 nm collagen fibril by 35% in the WT, but only by 25% in the *P2X_7_*^−/−^ stromas in comparison to undigested (Figure 6A,B). Furthermore, Chondroitinase ABC digested 74% of the filaments in WT, but only 55% of those in the *P2X_7_*^−/−^ group in comparison to undigested (Figure 6A,C). The digestion of KSPGs by Keratanase I showed equal distribution throughout the stroma of undigested filaments throughout both WT and *P2X_7_*^−/−^ corneas (Figure 6B). In contrast, digestion of SLRPs by Chondroitinase ABC revealed that the anterior portion of both WT and *P2X_7_*^−/−^ stromas was composed of a significantly higher concentration of chondroitin sulfate moieties than the posterior stroma (Figure 6C). When the tissue was digested with both Keratanase I and Chondroitinase ABC, 88% of the GAG filaments were removed in the WT, as opposed to 72% of those in the *P2X_7_*^−/−^ (Figure 6C). These results may indicate that the baseline level of Chondroitinase-undigested proteoglycans, or KSPGs and HSPGs, are reciprocally regulated in WT and *P2X_7_*^−/−^.

### Changes in localization of heparan sulfate proteoglycans

**Figure 7:**
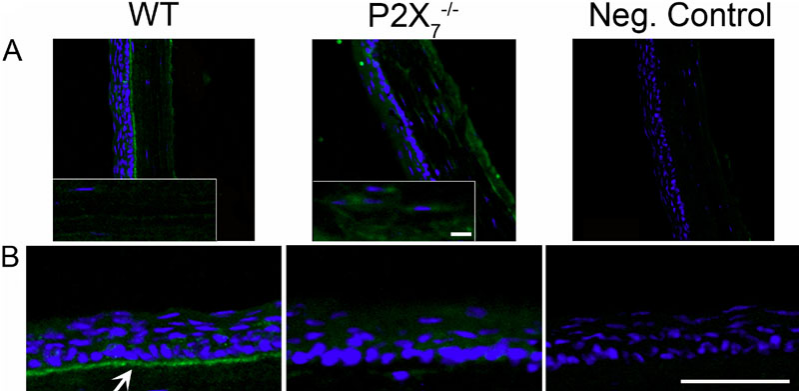
Perlecan localization is altered in *P2X_7_*^−/−^ stromas. Frozen corneas were sectioned and stained with antibody against perlecan, FITC-conjugated IgG, and To-Pro 3AM. Negative controls were incubated with non-immune IgG instead of primary antibody, and To-Pro 3AM. Images are representative of three independent experiments. **A**: Perlecan expression is increased throughout the stroma in *P2X_7_*^−/−^ corneas. Inset: enlarged regions from the central stroma with enhanced signal to show detail of localization. Scale bar: 10 μm. **B**: Perlecan is localized to the basement membrane in WT corneas (arrow) but not in *P2X_7_*^−/−^ corneas. Scale bar: 500 μm.

As described previously, perlecan has been detected in low levels in the intact stroma, but is increased with injury [18,30,31]. In the *P2X_7_*^−/−^ mouse, perlecan was localized throughout the stroma with the most intense staining in the posterior region (Figure 7A, and inset). The staining was more extensive than that detected in the WT. The increase is consistent with the increased perlecan mRNA expression in *P2X_7_*^−/−^ stromas (Figure 2). In addition, in the WT perlecan is present along the basement membrane of the corneal epithelium, as reported, where it is hypothesized to play a role in epithelial adhesion [24,34]. However, this typical staining pattern of expression along the basal lamina and Descemet’s membrane was absent in the *P2X_7_*^−/−^ corneas (Figure 7B). These indicate that the regulation is altered.

**Figure 8:**
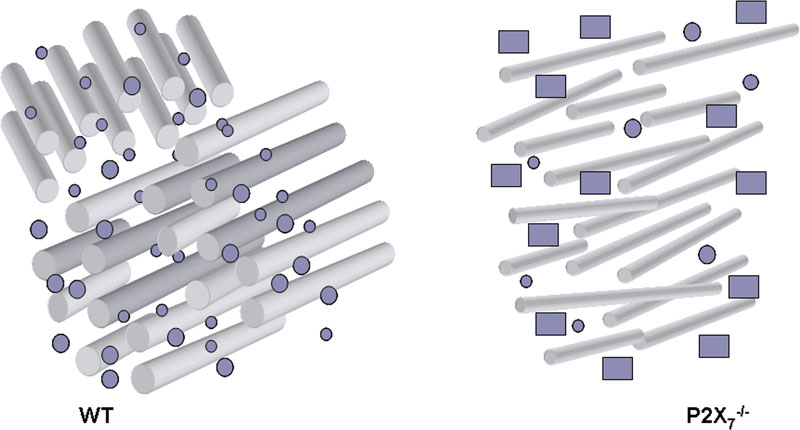
Schematic of WT and *P2X_7_*^−/−^ corneal stromas. Rods represent collagen fibrils, small circles represent SLRPs, and rectangles represent perlecan.

Based on our results, the schematic in Figure 8 summarizes the morphological differences in the *P2X_7_*^−/−^ cornea (Figure 8). In summary, we found that the P2X_7_ receptor regulates corneal stromal composition and organization at the transcriptional level of collagen, lysyl oxidase, and proteoglycan expression. In addition, this ionotropic receptor contributes to the proper structure and function of the cornea. In the absence of P2X_7_, collagen fibrils are thinner and more sparsely distributed, lacking the parallel fibril organization in perpendicular lamellar sheets. There are fewer SLRPs in *P2X_7_*^−/−^ stromas, and perlecan protein is present in *P2X_7_*^−/−^ but lacking in WT stromas.

## Discussion

These studies are the first to demonstrate that the ionotropic P2X_7_ receptor alters the expression and localization of proteoglycans in the corneal stroma. Previously, we showed that changes in regulation of collagen and proteoglycan expression in *P2X_7_*^−/−^ mice resulted in small fibril diameter, increased interfibrillar space and lamellar width, and overall decreased organization of collagen in the stroma [6]. The decrease in collagen α1(I) and collagen α3(V) mRNA correlates with the findings of decreased bone formation in *P2X_7_*^−/−^ mice [35]. Furthermore, perlecan has been implicated in long bone growth [34] and its expression was altered in the *P2X_7_*^−/−^ stroma. However, there is no known physical association of perlecan and collagen. Further studies may show links between P2X_7_, collagen deposition, and the role of perlecan in this process.

Additionally, we detected increased expression of the *LOX* mRNA transcript. Increased LOX activity is associated with decreased collagen fibril diameter and irregular arrangements of collagen in diabetic mice, where high glucose levels cause overexpression of *LOX*. This overexpression compromises the integrity of the extracellular matrix of the retina [8]. These data may explain our observation that *LOX* is significantly enhanced in *P2X_7_*^−/−^ stromas. Lysyl oxidase activity has a second function where it is regulated by hypoxia-inducible factors. It is in this function where LOX is enhanced in hypoxic tumors, promoting signaling through focal adhesion kinase, and thus contributing to the invasive properties of hypoxic cancer cells [36]. Future research may link these factors with a role in cell migration and wound healing.

Together, these data support our hypothesis that the downregulation of the P2X_7_ receptor “holds” the cornea in a pseudo-wounded state. Injury causes release of ligands such as transforming growth factor β (TGF-β) whose upregulation, in turn, increases the activity of LOX to expedite crosslinking of extracellular matrix components for wound healing [36,37]. Furthermore, the increased presence of type III collagen mRNA and the HSPGs syndecan 1 and perlecan mRNA in the *P2X_7_*^−/−^ corneal stroma is typical of the wounded phenotype [18,33,38]. This expression is often seen in stromal scars where type III collagen deposition and perlecan overexpression occur along with downregulation of SLRPs [33,38,39]. Perlecan, as previously mentioned, is found along the basement membranes of epithelium and endothelium [24,34]. Not only was this localization of perlecan lacking in *P2X_7_*^−/−^ corneas, but also expression of perlecan was enhanced throughout the posterior stroma, also indicative of injury [18,30,31]. Syndecan is important for keratinocyte activation, and syndecan-deficient mice have difficulty in wound healing and re-epithelialization after injury [25]. We observed increases in syndecan 1, perlecan, and type III collagen mRNA in unwounded *P2X_7_*^−/−^ corneal stromas. Taken together, these factors indicate that the lack of P2X_7_ maintains the cornea in a pseudo-wounded state.

The role of the P2X_7_ receptor in expression and localization of perlecan provides more detail into alterations that can occur at the interface of the corneal epithelium and basal lamina. The lack of perlecan in the basement membrane zone may contribute to the fragility of the tissue that was observed, however the fragility is not due to a decrease in the number of hemidesmosomes compared to WT [6]. Previous experiments showed separation in the anterior stroma in wounded *P2X_7_*^−/−^ corneas, rather than at the basal lamina, and we speculated that this was due to loose collagen fibrils in the anterior stroma. Observations made by second harmonic imaging show collagen fibrils inserting into Bowman’s membrane in normal corneas, which were absent in keratoconic mice [40]. Perhaps the change in matrix synthesis has altered mechanical constraints and fibrils are less organized anteriorly. The absence of perlecan at the basement membrane may have a larger effect on the collagen organization underneath the basement membrane than on the adhesion between the epithelium and the stroma, implying that there are other adhesive proteins at work at the basement membrane that may attach to loose collagen fibrils, pulling them with the epithelium during wounding.

The observed increase in biglycan mRNA transcripts was expected because of the observed decrease in decorin mRNA transcripts. Decorin knockout mice have shown a compensatory upregulation of biglycan, but the reverse was not observed in biglycan null mice [12,23]. Macroscopically, *P2X_7_*^−/−^ mice had no observable opacity [6], so the coordination between decorin and biglycan in collagen regulation may contribute to some maintenance of corneal transparency and function in *P2X_7_*^−/−^ mice. In contrast, previous experiments have shown decreases in keratocan expression in lumican null mice, implying that lumican plays a regulatory role for keratocan expression [41]. The compensatory mechanism observed in decorin null mice for biglycan is not observed in lumican null mice for keratocan, as shown in the *P2X_7_*^−/−^ mice. The role of these proteoglycans in corneal transparency is evident in the lumican knockout mice’s profoundly opaque corneas and altered collagen fibrillogenesis [16,17,41]. It is important to note that *P2X_7_*^−/−^ mice showed decreased levels of proteoglycans, rather than a complete lack of these proteoglycans, so the general transparency of *P2X_7_*^−/−^ corneas implies that proteoglycan expression is graded proportional to its function in organizing collagen in the stroma.

We conclude that the deficiency of P2X_7_ alters protein expression in the corneal stroma generating a morphology analogous to that seen in a wounded phenotype. P2X_7_ regulation at the transcriptional level of not only collagen itself, but also of proteins that aid in its organization in the corneal stroma, ultimately results in the altered stromal architecture seen in *P2X_7_*^−/−^ corneas.
